# Differential Pathways to Adult Metabolic Dysfunction following Poor Nutrition at Two Critical Developmental Periods in Sheep

**DOI:** 10.1371/journal.pone.0090994

**Published:** 2014-03-06

**Authors:** Kirsten R. Poore, Lisa J. Hollis, Robert J. S. Murray, Anna Warlow, Andrew Brewin, Laurence Fulford, Jane K. Cleal, Karen A. Lillycrop, Graham C. Burdge, Mark A. Hanson, Lucy R. Green

**Affiliations:** Academic Unit of Human Development and Health, Faculty of Medicine, University of Southampton, Southampton, United Kingdom; The University of Manchester, United Kingdom

## Abstract

Epidemiological and experimental studies suggest early nutrition has long-term effects on susceptibility to obesity, cardiovascular and metabolic diseases. Small and large animal models confirm the influence of different windows of sensitivity, from fetal to early postnatal life, on offspring phenotype. We showed previously that undernutrition in sheep either during the first month of gestation or immediately after weaning induces differential, sex-specific changes in adult metabolic and cardiovascular systems. The current study aims to determine metabolic and molecular changes that underlie differences in lipid and glucose metabolism induced by undernutrition during specific developmental periods in male and female sheep. Ewes received 100% (C) or 50% nutritional requirements (U) from 1–31 days gestation, and 100% thereafter. From weaning (12 weeks) to 25 weeks, offspring were then fed either *ad libitum* (CC, UC) or were undernourished (CU, UU) to reduce body weight to 85% of their individual target. From 25 weeks, all offspring were fed *ad libitum*. A cohort of late gestation fetuses were studied after receiving either 40% nutritional requirements (1–31 days gestation) or 50% nutritional requirements (104–127 days gestation). Post-weaning undernutrition increased *in vivo* insulin sensitivity, insulin receptor and glucose transporter 4 expression in muscle, and lowered hepatic methylation at the delta-like homolog 1/maternally expressed gene 3 imprinted cluster in adult females, but not males. Early gestational undernutrition induced lower hepatic expression of gluconeogenic factors in fetuses and reduced *in vivo* adipose tissue insulin sensitivity in adulthood. In males, undernutrition in early gestation increased adipose tissue lipid handling mechanisms (lipoprotein lipase, glucocorticoid receptor expression) and hepatic methylation within the imprinted control region of insulin-like growth factor 2 receptor in adulthood. Therefore, undernutrition during development induces changes in mechanisms of lipid and glucose metabolism which differ between tissues and sexes dependent on the period of nutritional restriction. Such changes may increase later life obesity and dyslipidaemia risk.

## Introduction

The worldwide incidence of obesity nearly doubled between 1980 and 2008, affecting both developed countries and developing societies that are undergoing socio-economic transition [1]. Strategies to reduce obesity by promoting healthy eating and an active lifestyle are producing disappointing results [2] and lifestyle factors do not appear to have the same impact on obesity in all individuals. Epidemiological and animal studies demonstrate that early life nutrition can exert a major influence on susceptibility to obesity, cardiovascular and metabolic diseases in adulthood [3], [4]. Thus, knowledge of an individual’s nutritional pathway during development may be beneficial in devising effective strategies to reduce the risk of obesity and its consequences.

The timing of nutritional challenges during development dictates the type, direction and magnitude of changes in the offspring’s metabolic and cardiovascular phenotype. Mid- and late-gestation exposure to the Dutch Famine reduced birth weight and glucose tolerance in adulthood, which worsened with obesity [5] while early gestation famine exposure did not affect birth weight, but increased coronary heart disease prevalence and atherogenic risk factors [6], [7]. Postnatally, nutrition and growth patterns also influence later metabolic health, with reduced birth size, persistent thinness in infancy but accelerated childhood growth and later obesity conferring increased risk of insulin resistance and dyslipidaemia [8]–[11]. Malnutrition in the first year also reduces glucose tolerance and insulin sensitivity in young men, independent of birth weight [12]. As well as investigating low birth weight and thinness at birth [13], [14] it is now clear that a range of possible developmental pathways to poor health in adulthood should be considered. These include the periconceptional and fetal periods and/or early postnatal infant life and beyond.

Animal models have confirmed the influence of different windows of sensitivity on offspring phenotype and work towards elucidating mechanisms in different tissues [15]–[20]. In sheep, undernutrition either during the first month of gestation or immediately after weaning (before puberty/adolescence) induces differential, sex-specific changes in metabolic and cardiovascular systems in adulthood. In males, cardiovascular abnormalities such as left ventricular hypertrophy were observed following exposure to undernutrition in early gestation (with no effect on birth weight) which was ameliorated if postnatal nutrition was relatively ‘matched’ to that prenatally [21]. In contrast, post-weaning undernutrition improved glucose tolerance and insulin sensitivity in both young and mature females, regardless of early gestation undernutrition [22]. Small for gestational age human infants also show increased sensitivity to insulin [23]. In females particularly, this may help return body weight towards a threshold necessary for successful reproductive function [23]. No such adaptation was observed in male sheep undernourished post-weaning [22], suggesting they must employ different strategies to regain body weight. Although an increase in fat was observed in young but not mature adult females exposed to both early gestation and post-weaning undernutrition [22], metabolic abnormalities may become apparent before overt obesity [24]. We suggest that enhanced insulin sensitivity in postnatally undernourished females may facilitate body weight recovery but increase their susceptibility to later obesity so long as nutrient supply is abundant. Indeed alterations in glucose metabolism and liver function in aged sheep exposed to maternal undernutrition have been observed only following a later period of unlimited feeding [25].

Epigenetic regulation by DNA methylation provides a mechanism linking suboptimal nutrition with long-lasting changes in specific gene expression and later phenotype [26], [27], [28]; for example epigenetic markers in neonatal blood and cord tissue have been linked with later adiposity and cardiometabolic risk factors [29], [30]. Furthermore, hepatic epigenetic modifications are detectable in early postnatal life following maternal undernutrition, before the phenotypic changes appear within the liver or other metabolic tissues, suggesting an interaction between the later environment and these modifications [24], [31]. The influence of early postnatal diet on epigenetic mechanisms remains little studied [32].

To develop biomarkers of risk and suggest preventative measures for obesity requires a detailed molecular phenotype in metabolically active tissues and understanding of lipid metabolism that may predispose to inappropriate adipose deposition. Large animal models allow links between specific molecular and epigenetic alterations in a range of tissues and whole animal phenotype. The aim of this study was therefore to determine underlying differences in lipid metabolism in male and female sheep following early gestation and/or post-weaning undernutrition, both *in vivo* and at the molecular level. We have measured circulating non-esterified fatty acid (NEFA) and triacylglycerol (TAG) during a glucose tolerance test (GTT) and mRNA levels of peroxisome proliferator-activated receptor γ (PPARγ; regulation of adipocyte differentiation), lipoprotein lipase (LPL; hydrolyses triglycerides and regulates their movement into adipocytes) and glucocorticoid receptor (GR; mediates glucocorticoid effects on adipose lipid accumulation and mobilisation) in metabolically active tissues. The mechanistic basis of the persistent, sex-specific effect of post-weaning undernutrition on metabolic homeostasis was also examined in tissues that regulate glucose and lipid uptake by measuring insulin receptor (IR), glucose transporter 4 (GLUT4), GR, phosphoenolpyruvate carboxykinase (PEPCK) and glucose-6-phosphatase (G6Pase). We have also investigated epigenetic modifications of hepatic genes regulating metabolic homeostasis and body composition by measuring methylation of imprinting control regions (ICRs) of the insulin-like growth factor (IGF) 2/H19 and insulin-like growth factor-2 receptor (IGF2R) imprinted clusters and of the delta-like homolog 1 (DLK1)/maternally expressed gene 3 (MEG3) imprinted cluster, which are involved in fetal and early postnatal growth and in adipocyte development, respectively, and have been linked with metabolic abnormalities and changes in adiposity [33]–[35]. We proposed that for females undernutrition prior to puberty will have a greater impact on mechanisms that control lipid and glucose metabolism, given the importance of maintaining adequate body condition for future reproductive success. In males, we proposed early gestation undernutrition will have a greater impact on mechanisms regulating adiposity, due to the relatively greater need for nutrients to drive their faster growth rate during gestation. We found that exposure to both gestational and post-weaning undernutrition induces sex- and tissue-specific effects on lipid and glucose handling mechanisms that may increase obesity and dyslipidaemia risk in later life.

## Materials and Methods

### Study of Adult Offspring Exposed to Early Life Undernutrition

#### Animals

All procedures were carried out in accordance with the regulations of the UK Home Office Animals (Scientific Procedures) Act, 1986 (PPL 30/1858). The data in this study comes from the same cohort of sheep previously studied by us and for which other data have been published elsewhere [21], [22], [36]. Fifty-nine Welsh Mountain ewes in their second or third parity (2–3 years old), carrying singleton and twin pregnancies, and of uniform good body condition score (BCS; ∼3 on a scale of 1–5 [37]) were used in this study. Full details about ewe mating, ewe and lamb housing and diets are presented elsewhere [22]. All manipulations of ewes and of lambs until 1.5 years of age took place at the Royal Veterinary College, North Mymms. A flow diagram showing the allocation of ewes and their offspring to the different dietary regimes is presented in Figure S1.

Before conception, ewes were randomly assigned to a control or a dietary restricted group. Control ewes (early gestation control, C; *n* = 29; 12 singleton (s), 17 twin (t)) received 100% nutritional requirements before and throughout gestation. From 1–31 days gestation (dGA; term = 147 days), ewes in the dietary restricted group (early gestation undernutrition, U; *n* = 31; 15 s, 15 t) received 50% nutritional requirements and then 100% requirements for the remainder of gestation. From −7 dGA, ewes were weighed weekly. The starting pelleted diet ration for each ewe was calculated using RUMNUT software (Ruminant Nutrition) version 5 for sheep (AT Chamberlain, Hampshire) based on initial body weight. This ration was adjusted weekly according to their weight measurement. The RUMNUT software was based on AFRC Guidelines for pregnant sheep [38] and incorporated adjustments for weight gain according to gestational age. Water was provided *ad libitum*. Ewes were individually penned from −7 to 37 dGA and group housed thereafter with animals at a similar gestational age. Ewes delivered and suckled lambs naturally while receiving 100% nutritional requirements, with further feed ration adjustments according to lactational needs [38].

A total of 77 male and female, singleton and twin, lambs were included in the study (Figure S1). Lambs were weaned at 12 weeks, after gradual introduction of the post-weaning diet [22]. Lambs from C and U ewes were grouped with approximately 10 others of similar body weight and post-weaning treatment group in open barns. For each lamb, an individual linear weight trajectory was calculated for the period 12–25 weeks that was based on weights taken at birth, 4, 8 and 12 weeks. Lambs were assigned to either a post-weaning control diet (yielding early gestation control/post-weaning control, CC; early gestation undernutrition/post-weaning control, UC) or post-weaning undernutrition diet (early gestation control/post-weaning undernutrition, CU; early gestation undernutrition/post-weaning undernutrition, UU). Post-weaning control lambs were fed 100% nutritional requirements to follow their individual trajectory from 12–25 weeks and post-weaning undernutrition lambs were fed a restricted pelleted diet such that body weight was reduced to 85% of their individual target weight from 12–25 weeks. Lambs had free access to hay throughout. To keep body weight on the desired trajectory, lambs were monitored individually by weekly weighing and feed ration adjustment. If necessary, lambs were temporarily removed to individual pens to maintain body weight at the desired level. For twin lambs, one twin was assigned to the post-weaning control diet and the other to the post-weaning undernutrition diet, except for twin pairs from 14 ewes in which only one offspring of the pair was used (Figure S1). The number, sex and proportion of singleton to twin lambs in each group are as follows; males: CC, *n* = 13 (5 s, 8 t), CU, *n* = 8 (3 s, 5 t), UC, *n* = 8 (4 s, 4 t), UU, *n* = 11 (5 s, 6 t); females: CC, *n* = 9 (2 s, 7 t), CU, *n* = 7 (2 s, 5 t), UC, *n* = 10 (2 s, 8 t), UU, *n* = 11 (4 s, 7 t).

From 25 week onwards, all lambs were returned to larger group housing and received 100% nutritional requirements. All lambs were weighed at 35 weeks and just prior to experiments (see below) at 16.5±0.1 months (1.5 years) and 29.6±0.2 months (2.5 years) of age. At each experimental age, body condition score was assessed manually and fat and muscle depths were measured by ultrasound in the third lumbar region by a small number of experienced animal technicians, as described previously [22].

For technical reasons e.g catheter loss prior to *in vivo* experiments or difficulty with laboratory procedures, data from the full number of animals in each group were not always achieved. The number of observations for each data set is indicated in the legend of each table or figure or within the text of the results section.

#### Surgical techniques

As described previously [22], surgery at 9.9 months (to create carotid artery loops for repeated arterial access and to vasectomise male lambs) and experiments at 1.5 years of age were performed at the Royal Veterinary College and experiments at 2.5 years were performed at the University of Southampton. Prior to each set of experiments, sheep were moved into individual cages and acclimatised for 4–6 days, with no change in their feeding regime. Indwelling carotid artery and jugular vein catheters were inserted under general anaesthesia (at 1.5 years: 2–4% halothane in O_2_ by face mask; at 2.5 years: induced by thiopentone sodium (10 mg.kg^−1^, *i.v.*) and maintained by 2–4% halothane in O_2_ by endotracheal tube). Antibiotic treatment (15 mg.kg^−1^, *i.m*.; Betamox) was administered to all sheep. At 1.5 years, catheters were maintained for 3 days then removed prior to sheep returning to group housing. At 2.5 years, catheters were maintained for up to 17 days during a series of experiments and subsequent *post mortem* that are described elsewhere [21], [22], [39].

#### Experimental protocol

At 1.5 and 2.5 years of age, a glucose tolerance test (GTT) was performed following an overnight fast (16.00 h–09.00 h). Glucose (0.5 g.kg body weight^−1^) was administered as an intravenous bolus over 3 minutes and arterial blood samples (3 ml into chilled EDTA tubes) were collected immediately before (0 min) and at 10, 20, 30, 60, and 120 min after the start of the glucose administration (time 0). All blood samples were centrifuged immediately (10 min at 4°C) and plasma was stored at –20°C for later laboratory analysis.

### Study of Fetuses Exposed to Gestational Undernutrition

#### Animals

A separate cohort of 23 Welsh Mountain primiparous ewes, carrying singleton fetuses, of uniform body condition score were used for collection of late gestation fetal tissues. As described previously [40], ewes were randomly assigned to a control or one of two dietary restricted groups, housed individually on straw and from −16 dGA (adjusted to gestational age) were fed a complete pelleted diet (89.2% dry matter as fed, providing 10.7 MJ.(kg dry matter)^−1^ (metabolisable energy) and 14.8% protein) with water available *ad libitum*. Control ewes (C, *n* = 8; 4 male and 4 female fetuses) were fed 100% nutritional requirements throughout gestation. Early gestation nutrient-restricted ewes (E, *n* = 9; 5 male and 4 female fetuses) were fed 40% of nutrient requirements from 1−31 dGA and late gestation nutrient-restricted ewes (L, *n* = 6; 4 male and 2 female fetuses) were fed 50% of nutrient requirements from 104−127 dGA (term ∼147 dGA). At all other times, ewes received 100% nutrient requirements. Full details about ewe mating, surgical and experimental procedures in these fetuses that are not presented in the current study are described elsewhere [40], [41].

### Post Mortem Procedures

At the completion of all experimental protocols, *i.v.* pentobarbitone sodium overdose (200 mg.ml^−1^ Pentoject, Animalcare Ltd, UK) was used to kill adult sheep (2.5 years) or pregnant ewes and their fetuses (at 127 dGA). Samples of left and right lobes of the liver were removed from both adults and fetuses and skeletal (flexor digitorum longus) muscle and perirenal adipose tissue was removed from adults. All tissues were snap frozen in liquid nitrogen and stored at −80°C.

### Laboratory Analysis

#### mRNA expression analysis

Total RNA was extracted from adult muscle, adult adipose tissue and fetal and adult left and right liver lobe using the TRIzol method and reverse transcribed into cDNA. Real-time RT PCR (ABL Prism 7700 and 7500 Sequence Detection System, Applied Biosystems Step One Plus) was used to measure mRNA expression for IR in all adult and fetal tissues, GLUT4 in adult muscle and adipose tissue, GR in adult adipose tissue and adult and fetal liver, LPL and PPAR-γ in adult adipose tissue, PEPCK and G6Pase in adult and fetal liver, IGFII and IGF2R in the left lobe of adult liver. Primers and probes (Table S1) were designed using Primer Express Software (Applied Biosystems, USA). GR in adult adipose tissue, IGFII and IGF2R gene expression and expression of housekeeping genes were measured using SYBR Green (JumpStart Taq ReadyMix, Sigma Aldrich S4438). Gene expression in adult muscle and adipose tissues was normalised to the geometric mean of β-actin and glyceraldehyde 3-phosphate dehydrogenase (GAPDH) mRNA expression, as determined using a GeNorm normalising kit (Primer Design, Southampton, UK) validated for use in the sheep. In adult and fetal liver, gene expression was normalised to the geometric mean of glyceraldehyde 3-phosphate dehydrogenase (GAPDH), ribosomal protein 19 large subunit (RPL-19) and β-actin mRNA expression, as determined using the GeNorm normalising kit. GR gene expression in adult adipose tissue was performed before the GeNorm kit became available so these data were normalised to 18S mRNA expression. Coefficients of variation ranged from 6% to 10%.

#### PCR-based methylation analysis

PCR was used to amplify regions within the imprinting control regions (ICRs) of the DLK1/MEG3, IGF2R and IGF2/H19 imprinted clusters (Table S1) then DNA methylation levels were analysed by pyrosequencing using a PyroMark Q96 (Biotage/Qiagen), as described previously [42]. In brief, genomic DNA from adult left liver lobe was bisulfite converted using the EZ DNA Methylation-Gold Kit (Zymo Research), following manufacturer’s guidelines. The expression of the DLK1/MEG3 imprinted cluster is regulated, like the IGF2/H19 cluster, by binding of CTCF within the ICR region, which is itself influenced by the presence or absence of DNA methylation at CTCF binding sites. We identified a potential CTCF site within the MEG3 ICR, based upon reported CTCF consensus sequences [43]–[45] that contained CpG dinucleotides 2, 3, and 4, and was directly adjacent to CpG1 within the pyrosequencing assay. Methylation at the H19 loci was examined across CTCF binding site IV, which has previously been shown to regulate H19 expression [44], [46]. Methylation within the ICR of IGF2R, a 2.3 Kb CpG island regulates IGF2R expression in sheep [46], [47].

Determination of DNA methylation within the GR promoter region was undertaken prior to the availability of the above methodology, therefore for this data set methylation-sensitive PCR was used. PCR was used to amplify DNA extracted from adult left and right liver lobes and treated with methylation-sensitive restriction enzymes (Acil and Hpall). GR primers were designed to amplify two regions within the CpG island within the GR promoter region, based on a partial ovine sequence determined by express sequence tag mapping to the bovine sequence (Table S1).

#### Plasma lipid analysis

Plasma NEFA and TAG concentrations during GTT at 1.5 and 2.5 years were measured to assess insulin-induced inhibition of lipolysis in adipose tissue. A randomly chosen subset of animals was included for these measurements. Plasma NEFA concentrations were measured by a spectrophotometric two-stage enzymatic assay kit (Wako NEFA HR2, ACS-ACOD method; Alpha Laboratories, Eastleigh, UK) on a Konelab 20 autoanalyser (Thermo Fisher Scientific, Hemel Hempstead, UK). This method was not sensitive enough to measure TAG concentrations. Therefore, gas chromatography (HP 6890 Series GC System equipped with a flame ionisation detector and 7683B Series Injector) was used, following separation of lipid classes by solid phase extraction, to measure plasma TAG concentrations (extracted from 0.4 g plasma, made up to 0.8 ml) or adipose total lipid content (extracted from 20 mg adipose tissue), as described previously [48].

### Data Analysis

For NEFA and TAG analysis during GTT in adult sheep, summary measures (baseline, slope, maximum fall from baseline and area under curve relative to baseline (AUC)) were calculated. For all adult data except liver, the effects of four main factors were tested (early gestation diet, post-weaning diet, sex and offspring number (i.e. singleton or twin)) using multifactorial analyses of variance (4-way ANOVA). For adult liver, the same factors were examined using multifactorial analyses of variance for repeated measures (RM ANOVA), with liver lobe as the repeated measure. For fetal liver, the main factors of maternal dietary group and fetal sex were used in a RM ANOVA, with liver lobe as the repeated measure. Where significant effects of a main factor or interactions between the main factors were found, further analyses were performed within subsets of the data. Linear regression analysis was used to examine relationships between two factors: this was done within each sex, across all nutritional groups. Statistical analyses were performed using GraphPad Prism version 5 and SPSS version 11. Data are expressed as mean ± S.E.M. and significance was accepted at p<0.05, with a trend noted when 0.05<p<0.1.

## Results

### Lipid Metabolism during Glucose Tolerance Test

#### Diminished insulin-induced inhibition of lipolysis in adult males and females undernourished in early gestation

**Figure 1:**
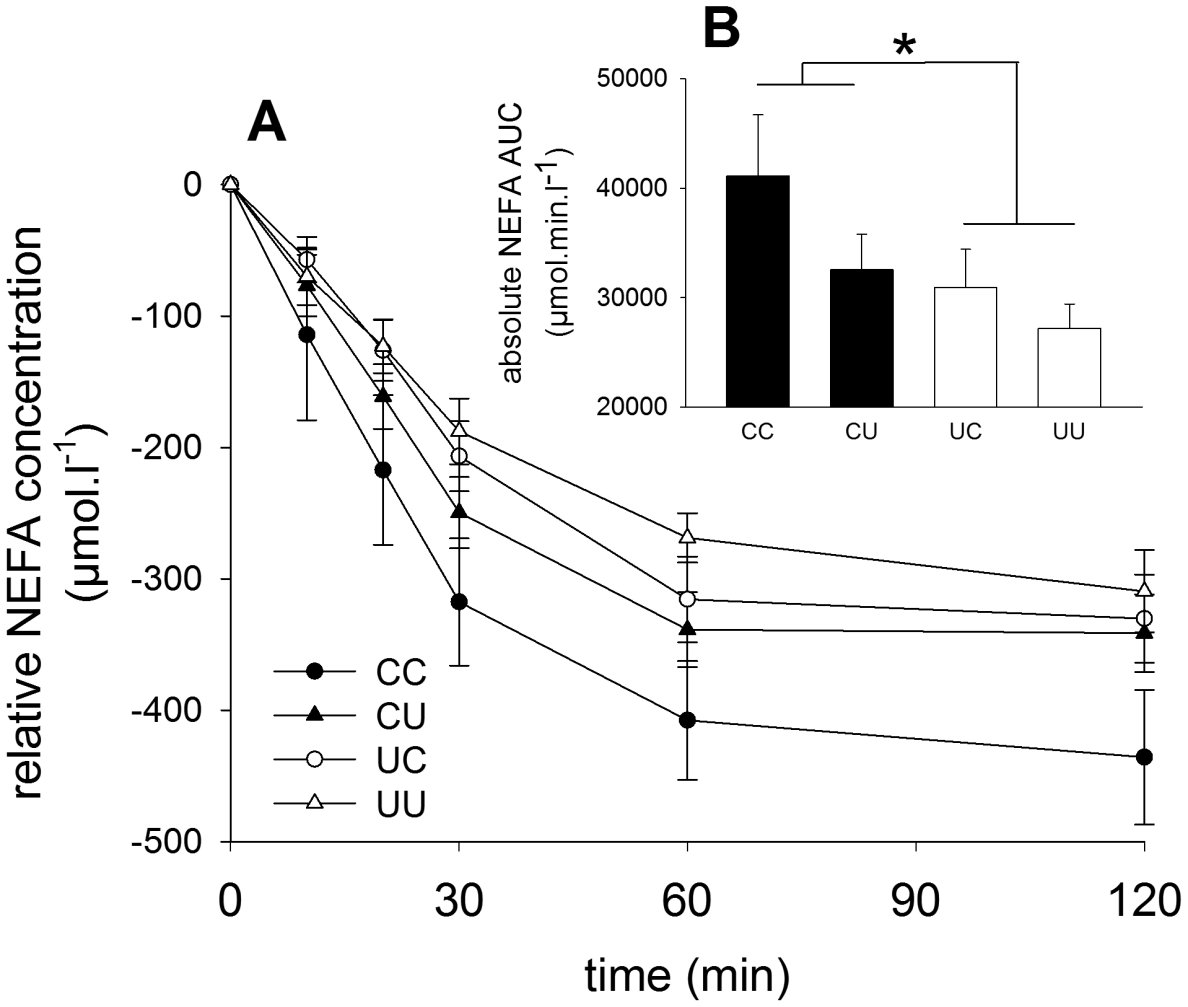
*In vivo* lipid handling in 2.5 year-old sheep. (A) Plasma non-esterified fatty acid (NEFA) concentrations relative to baseline in male and female sheep combined during a glucose tolerance test at 2.5 years. Glucose (0.5 g.kg^−1^) was given at time 0. (B) Absolute area under the NEFA response curve (AUC); *p<0.05, early gestation undernutrition groups less than early gestation control groups. CC: *n* = 13; CU: *n* = 14; UC: *n* = 15; UU: *n* = 13). Values are mean ± S.E.M.

Plasma NEFA and TAG concentrations during a glucose tolerance test (GTT), performed in both young (1.5 years) and mature (2.5 years) adulthood [22] were determined as a surrogate measure of insulin-induced adipose tissue lipid metabolism. The insulin-induced fall in NEFA output during GTT was reduced (p<0.05) at 2.5 years in sheep exposed to early gestation undernutrition, regardless of post-weaning nutrition (Fig. 1B). There was no effect of sex on this response. The slope from 10–30 min during the GTT tended to be shallower in UC and UU groups (−9.4±0.9 *vs.* −7.2±1.4 µmol.l.min^−1^, p<0.1). Basal NEFA and TAG concentrations at 2.5 years were not influenced by early life nutrition (Table 1). There was no effect of early gestation undernutrition on plasma NEFA and TAG concentrations during the GTT at 1.5 years. Faster relative growth during the first 12 postnatal week was associated with reduced NEFA AUC at 2.5 years (r^2^ = 0.20, p<0.05). Low birth weight was associated with increased NEFA AUC at 2.5 years (r^2^ = 0.16, p<0.005).

**Table 1:** Basal plasma NEFA and TAG concentrations in young (1.5 years) and mature (2.5 years) adult male and female sheep.

	Males				Females			
	CC	CU	UC	UU	CC	UC	CU	UU
1.5 Years:								
NEFA (µmol.l^−1^)	792±70^(5)^	647±53^(5)^	747±104^(5)^	748±132^(5)^	828±93^(6)^	914±72^(5)^	907±52^(5)^	815±90^(5)^
TAG (µmol.l^−1^)	36.4±4.0^(6)^	27.1±6.5^(4)^	5.3±5.3^(6)^	34.9±11.6^(6)^	38.2±11.4^(5)^	38.6±4.9^(5)^	33.9±4.0^(5)^	41.5±12.0^(5)^
2.5 Years:								
NEFA (µmol.l^−1^)	482±65^(7)^	410±35^(8)^	375±52^(8)^	384±46^(8)^	544±83^(6)^	448±49^(6)^	443±52^(7)^	399±50^(6)^
TAG (µmol.l^−1^)	22.8±3.2^(9)^	30.8±6.7^(6)^	19.0±1.8^(6)^	27.3±5.0^(7)^	25.0±2.3^(6)^	29.9±7.5^(5)^	17.4±3.2^(7)^	21.3±1.6^(6)^

Data are mean ± S.E.M. Values in brackets represent number of observations.

#### Increased insulin sensitivity in mature adult females undernourished post-weaning

At 1.5 years, there were no overall effects of post-weaning nutrition or sex on plasma NEFA or TAG concentrations before (Table 1) or during GTT (data not shown). Basal NEFA and TAG concentrations in 1.5 year-old males and females combined were positively related to current fatness (back fat depth corrected for body weight; r^2^ = 0.16 and 0.19, respectively; p<0.01). At 2.5 years, in females only, the maximum fall in plasma TAG concentrations during GTT was greater (indicating increased insulin sensitivity) in animals undernourished only in the post-weaning period (CU, 11.5±5.5 µg.ml^−1^) compared to controls (CC, 8.7±1.4 µg.ml^−1^). Adipose tissue lipid content was also reduced (p<0.05) in CU compared to CC females at 2.5 years (Table 2).

**Table 2:** Genes of interest in liver and adipose tissue of mature (2.5 years) adult male and female sheep.

	Males				Females			
	CC	CU	UC	UU	CC	CU	UC	UU
	*n* = 13	*n* = 7	*n* = 8	*n* = 11	*n* = 9	*n* = 7	*n* = 9	*n* = 11
Liver – left lobe:								
GR mRNA^x^^,^ z	3.41±0.49	2.85±0.40	3.41±0.40	3.35±0.49	2.73±0.37	2.20±0.29	2.32±0.14	2.37±0.24
IR mRNA	0.187±0.023	0.222±0.066	0.304±0.081	0.282±0.056	0.268±0.030	0.203±0.025	0.276±0.041	0.228±0.041
PEPCK mRNA^y^^,^ z	0.258±0.049	0.190±0.021	0.313±0.033	0.294±0.055	0.166±0.036	0.194±0.038	0.224±0.041	0.140±0.027
G6Pase mRNA	0.485±0.051	0.521±0.116	0.463±0.059	0.522±0.100	0.536±0.084	0.486±0.078	0.471±0.078	0.455±0.060
GR methylation	0.013±0.004	0.021±0.012	0.010±0.002	0.018±0.004	0.013±0.004	0.011±0.005	0.049±0.043	0.013±0.004
IGFII mRNA	0.512±0.076	0.523±0.097	0.552±0.136	0.513±0.056	0.380±0.059	0.405±0.041	0.378±0.067	0.426±0.041
IGF2R mRNA	0.015±0.006	0.006±0.002	0.023±0.011	0.017±0.006	0.062±0.056	0.011±0.003	0.017±0.001	0.040±0.023
Liver – right lobe:								
GR mRNA^x^	7.18±0.67	7.57±0.66	6.68±0.94	7.42±1.44	3.67±0.56	3.51±0.30	3.75±0.60	3.77±0.47
IR mRNA	0.234±0.041	0.302±0.050	0.238±0.064	0.266±0.039	0.203±0.017	0.217±0.024	0.250±0.051	0.242±0.019
PEPCK mRNA	0.198±0.029	0.219±0.046	0.307±0.072	0.182±0.027	0.106±0.008	0.150±0.025	0.126±0.017	0.141±0.021
G6Pase mRNA	0.512±0.076	0.523±0.097	0.552±0.136	0.513±0.056	0.380±0.059	0.405±0.041	0.378±0.067	0.426±0.041
GR DNA methylation	0.015±0.006	0.006±0.002	0.023±0.011	0.017±0.006	0.062±0.056	0.011±0.003	0.017±0.001	0.040±0.023
Adipose tissue:								
IR mRNA	1.63±0.15	1.75±0.25	1.56±0.17	1.29±0.10	1.55±0.24	1.37±0.19	1.54±0.19	1.35±0.18
GLUT4 mRNA	1.55±0.16	1.66±0.12	1.38±0.18	1.51±0.13	1.06±0.18	1.28±0.18	1.42±0.15	1.49±0.32
PPARγ mRNA	1.85±0.17	1.83±0.24	1.82±0.15	1.68±0.15	0.97±0.16	0.88±0.11	0.96±0.09	0.97±0.14
Lipid content (mmol. mg^−1^)	507±27	500±34	514±15	491±37	567±14^a^	500±34^b^	535±14	551±14

Data are mean ± s.e.m.

a, b significant difference (p<0.05) between CU and CC groups in females;

x significant difference (p<0.001) between left and right liver lobes;

y significant difference (p<0.05) between left and right liver lobes in males;

z significant difference (p<0.001) between males and females: see text for more details. Values given have arbitrary units and are normalised to geometric means of β-actin, GAPDH and RPL-19 (liver) or β actin and GAPDH (adipose tissue).

### Tissue Expression of Factors Involved in Lipid Metabolism

#### Increased adipose tissue lipid handling factors in adult males undernourished in early gestation

**Figure 2:**
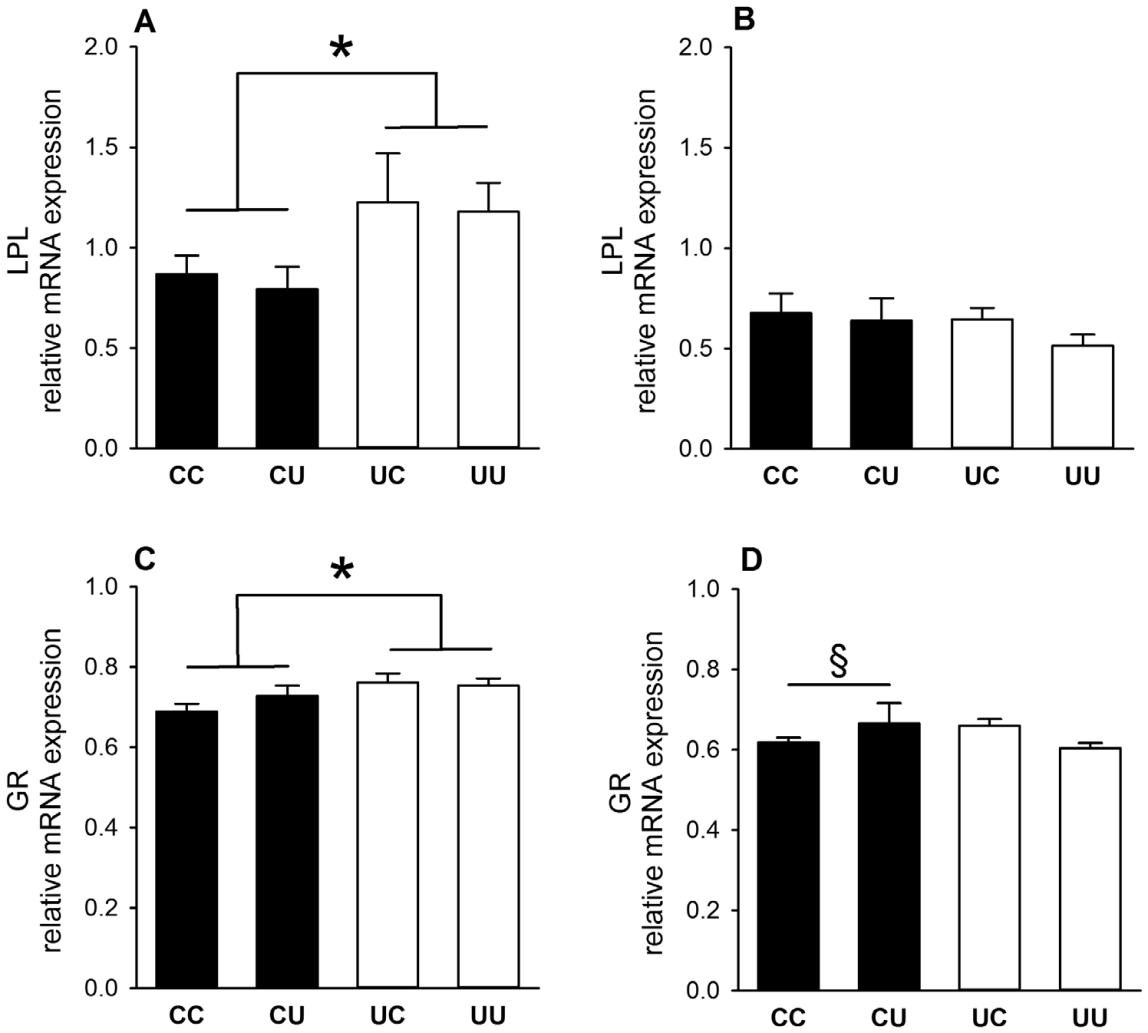
Factors involved in lipid handling in adipose tissue of 2.5 year-old sheep. Messenger RNA levels of lipoprotein lipase (LPL, normalised to geometric mean of β actin and GAPDH) and glucocorticoid receptor (GR, normalised to 18S) from male (A, C) and female (B, D) sheep (CC: male, *n* = 13, female, *n* = 9; CU: male, *n* = 7, female *n* = 7; UC: male, *n* = 8, female, *n* = 9; UU: male, *n* = 11, female, *n* = 11). Values are mean ± S.E.M. *p<0.05, early gestation undernutrition groups greater than early gestation control groups. § p = 0.08, CU greater than CC.

In males, but not females, both LPL and GR mRNA levels in adipose tissue at 2.5 years were significantly higher in sheep exposed to early gestation undernutrition (Fig. 2). This was not associated with an effect on adipose tissue lipid content (Table 2). PPARγ (Table 2), LPL (Fig. 2) and GR mRNA (1.49±0.05 *vs.* 0.97±0.05 arbitrary units) levels in adipose tissue at 2.5 years were significantly (p<0.001) higher in males than females. Faster relative growth during the first 12 postnatal weeks was associated with higher GR mRNA levels in adipose tissue (r^2^ = 0.11, p<0.05).

#### Adipose tissue factors are unaffected by post-weaning undernutrition

There were no significant effects of post-weaning nutrient restriction on mRNA levels of PPARγ, LPL or GR in adipose tissue of either male or female sheep at 2.5 years (Fig. 2, Table 2).

### Tissue Expression of Factors Involved in Glucose Metabolism

#### Hepatic gluconeogenic gene expression during fetal but not adult life is reduced by early and late gestation undernutrition

**Figure 3:**
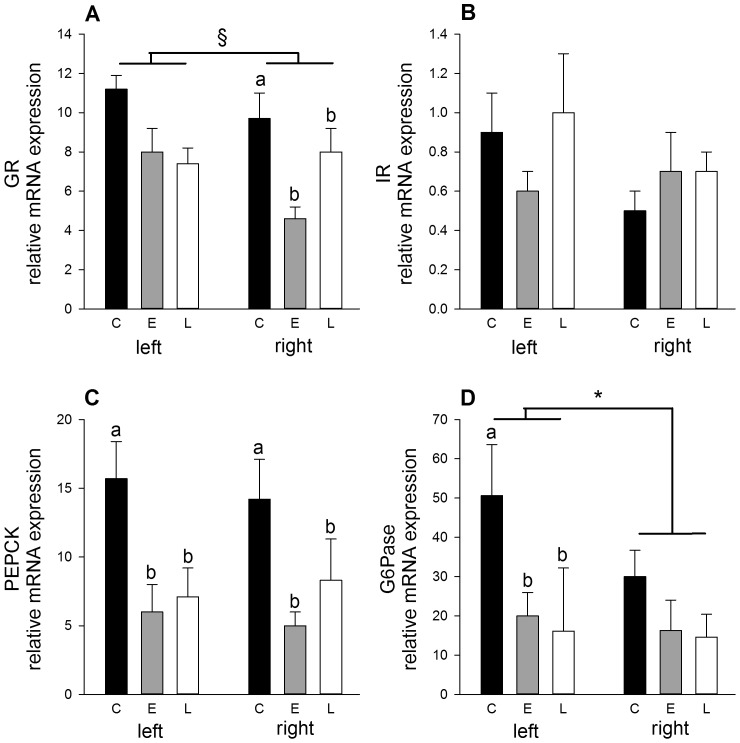
Factors involved in glucose homeostasis in liver of 127 dGA fetal sheep. Messenger RNA levels (normalised to geometric mean of β actin, GAPDH and RPL-19) of glucocorticoid receptor (GR; A), insulin receptor (IR; B), phosphoenolpyruvate carboxykinase (PEPCK; C) and glucose-6-phosphatase (G6Pase; D) in left and right liver lobes of control (C, *n* = 8), early gestation undernutrition (E, *n* = 9) and late gestation undernutrition (L, *n* = 6) fetuses (males and females combined). Values are mean ± S.E.M. Different letters denote significantly different values based on nutritional group (p<0.05). *p<0.01, § p = 0.065; difference between left and right liver lobes.

In a cohort of late gestation (127 days) singleton fetuses exposed to a 40% nutrient restriction in early gestation (compared to 50% in the adult cohort), PEPCK mRNA levels were reduced (p<0.05) in both hepatic lobes (Fig. 3). Levels of GR (p<0.01) and G6Pase (p<0.05) mRNA in fetal liver were also reduced by early gestation undernutrition, but in a lobe-specific manner. PEPCK, GR and G6Pase mRNA levels were similarly reduced in fetuses exposed during late gestation (104–127 days) (Fig. 3).

**Figure 4:**
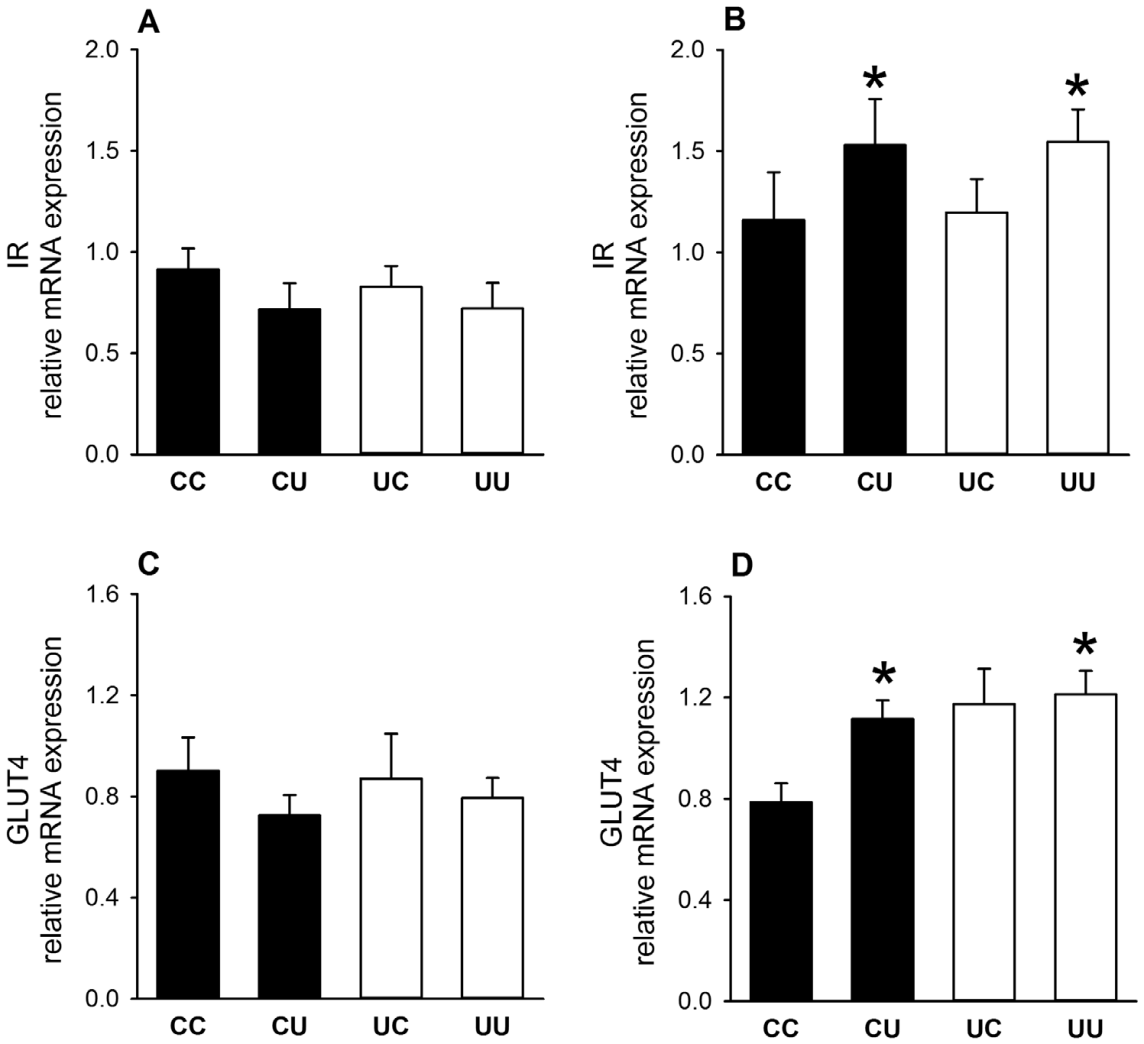
Components of insulin signalling in skeletal muscle of 2.5 year-old sheep. Messenger RNA levels (normalised to β actin and GAPDH) of insulin receptor (IR) and glucose transporter 4 (GLUT4) from male (A, C) and female (B, D) sheep (CC: male, *n* = 13, female, *n* = 8; CU: male, *n* = 7, female *n* = 7; UC: male, *n* = 6, female, *n* = 7; UU: male, *n* = 11, female, *n* = 10). Values are mean ± S.E.M. *p<0.05, post-weaning undernutrition groups greater than post-weaning control groups.

In adult tissues, early gestation undernutrition had no effect on IR mRNA levels in muscle (Fig. 4), adipose tissue or liver (Table 2), or on hepatic GR, PEPCK or G6Pase mRNA levels (Fig. 2, Table 2). While being a twin had no bearing on adult glucose tolerance in this model, GLUT4 mRNA levels in muscle were elevated in adult female twins undernourished in early gestation (UC and UU combined (*n* = 11), 1.28±0.08 *vs.* CC and CU combined (*n* = 12), 0.90±0.08 arbitrary units; p<0.005). Early gestation undernutrition increased PEPCK mRNA levels (p<0.05) in the adult right liver lobe compared to controls, but only in singleton males (0.31±0.04 *vs.* 0.18±0.04 arbitrary units).

#### Gluconeogenic genes in liver differs between the lobes

There was differential expression of gluconeogenic genes between the fetal liver lobes, with G6Pase (p<0.01) and GR (p = 0.065) expression overall greater in the left (29.09±5.93 and 8.85±0.66, respectively) compared to the right (20.34±4.15 and 7.18±0.73 arbitrary units, respectively) liver lobe. Regardless of early gestation or post-weaning undernutrition, in adult liver GR mRNA levels overall were greatest (p<0.001) in the right lobe compared to left lobe in males and females (males: 7.14±0.53 *vs.* 3.24±0.23; females: 3.56±0.25 *vs.* 2.30±0.16 arbitrary units) and PEPCK mRNA levels were greatest (p<0.05) in the left lobe compared to right lobe in males (0.27±0.02 *vs.* 0.22±0.02 arbitrary units). Both GR and PEPCK mRNA levels overall were higher (p<0.001) in adult male than female liver, regardless of lobe (5.19±0.22 *vs.* 2.93±0.26 and 0.24±0.02 *vs.* 0.14±0.02 arbitrary units, respectively) and in female twins compared to singletons (3.89±0.26 *vs.* 3.24±0.43 arbitrary units (right liver lobe) and 0.21±0.02 *vs.* 0.10±0.03 (left liver lobe), respectively, see also Table 2). DNA methylation within the GR promoter region in adult liver was not affected by lobe, nutrition, sex or offspring number (Table 2).

#### Increased insulin signalling factors in muscle in adult females undernourished post-weaning

Post-weaning undernutrition in adult female, but not male sheep, regardless of early gestation undernutrition, significantly (p<0.05) increased mRNA levels of IR and GLUT4 in skeletal muscle but not in peri-renal adipose tissue (Fig. 4, Table 2). Reduced growth rate during the period of post-weaning undernutrition from 12–25 weeks was also directly associated with increased IR and GLUT4 mRNA levels in muscle from females (r^2^ = 0.17, p<0.05 and r^2^ = 0.22, p<0.01, respectively). The effect of post-weaning undernutrition on GLUT4 mRNA levels was strongest in singleton sheep, as revealed by investigation of a significant (p<0.05) interaction between post-weaning undernutrition and offspring number: CU and UU singletons combined, 1.25±0.10 (*n* = 4) *vs.* CC and UC singletons combined, 0.74±0.12 (*n* = 4) arbitrary units. Post-weaning nutrition restriction had no effect in the liver on IR, GR, PEPCK or G6Pase mRNA levels (Table 2), except in the right liver lobe where PEPCK mRNA levels in singleton male sheep were reduced by post-weaning undernutrition (CU and UU combined, 0.16±0.02 (*n* = 8) *vs.* CC and UC combined, 0.31±0.06 (*n* = 10) arbitrary units).

### Hepatic Methylation Pattern of Factors Regulating Metabolic Homeostasis and Body Composition

#### Increased methylation of imprinted genes regulating growth in adult males undernourished in early gestation

**Figure 5:**
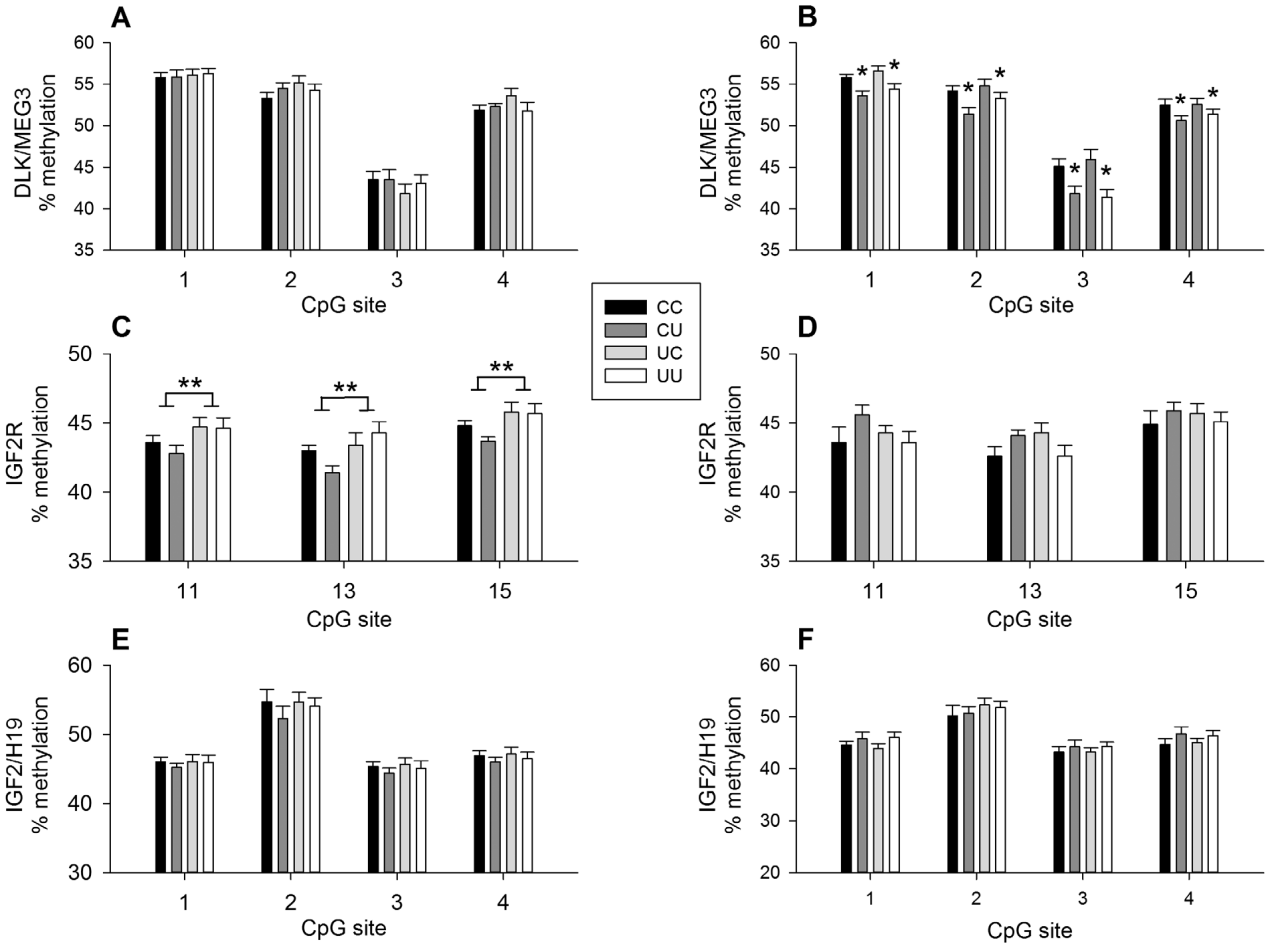
Epigenetic modification of genes regulating metabolic homeostasis and body composition in liver of 2.5 year-old sheep. Methylation of CpG dinucleotides within the ICRs of the DLK1/MEG3 (A, B), IGF2R (C, D) and IGF2/H19 (E, F) imprinted regions in male (A, C, E) and female (B, D, F) sheep (CC: male, *n* = 11, female, *n* = 9; CU: male, *n* = 7, female *n* = 7; UC: male, *n* = 8, female, *n* = 9; UU: male, *n* = 10, female, *n* = 11). Values are mean ± S.E.M. *p<0.05, post-weaning undernutrition less than post-weaning control; **p<0.05, early gestation undernutrition greater than early gestation control.

There was a significant (p<0.05) increase in methylation within the ICR of IGF2R at CpG 11, 13 and 15 in left liver lobe from 2.5 year-old males undernourished in early gestation but not from females (Fig. 5). Early gestation undernutrition had no effect on methylation at the IGF2/H19 or DLK/MEG3 loci in adult liver in either sex (Fig. 5). There were no effects of early gestation undernutrition on IGFII or IGF2R mRNA levels in adult liver (Table 2).

#### Decreased methylation of adipocyte development factor DLK/MEG3 in adult females undernourished post-weaning

In female but not male sheep, post-weaning undernutrition significantly reduced hepatic DLK/MEG3 methylation at CpG dinucleotides 1, 2, 3, and 4 (Fig. 5), an effect that could reduce expression of DLK1. There was no significant effect of post-weaning undernutrition on IGF2R or IGF2/H19 imprinted clusters (Fig. 5). Poor growth in females between 12 and 25 weeks was associated with decreased DLK/MEG3 methylation at CpG 2, 3 and 4 (r^2^ = 0.24, 0.29 and 0.18, respectively, p<0.005). There was a positive relationship in 2.5 year-old males between DLK/MEG3 methylation at CpG 4 in left liver lobe and muscle depth (r^2^ = 0.13, p<0.05).

## Discussion

This large animal study is unique in its comparison of the effects of a postnatal nutrient restriction challenge in the context of a previous prenatal nutritional challenge. We have shown that exposure to both early gestation and post-weaning undernutrition have sex- and tissue-specific effects on mechanisms of lipid and glucose metabolism, effects that may increase the risk of obesity and dyslipidaemia in later life. The presence of sex differences in the current study confirms differential effects on metabolic and cardiovascular parameters previously reported in sheep [21], [22], and goes further to suggest that males and females adopt different strategies in the face of reduced nutrition at critical windows of development.

We showed previously that female sheep undernourished in the post-weaning period make metabolic adaptations, improving their glucose handling, that persist from young through to mature adulthood [22]. In the current study, we show that this effect is likely brought about by enhanced insulin-induced glucose uptake specifically into muscle, via increased insulin receptor and glucose transporter 4 mRNA levels. Factors which are known to be involved in hepatic insulin sensitivity, in the regulation of gluconeogenesis and in adipose tissue differentiation and lipid hydrolysis, were unaffected by post-weaning undernutrition in this study. The effect in muscle is consistent with studies of improved insulin signalling capacity in intra-uterine growth restricted fetal sheep [49] and in rat offspring exposed to maternal low protein diet [19], and provides a mechanism whereby post-weaning undernutrition in females induces adaptations in muscle that enhance nutrient uptake and storage. If this effect was initiated shortly after the post-weaning challenge, prior to puberty, it may provide a strategy to facilitate the return to control body weight observed by 1.5 years (young adulthood) [22], which could be critical to ensure reproductive success. However with ageing and/or an over-abundant nutrient environment, these mechanisms may lead to inappropriate effects on body composition or fatness and metabolic homeostasis [25]. Indeed in human infants, a transition from enhanced insulin sensitivity during rapid early weight gain to insulin resistance has been observed, in association with a propensity for central fat accumulation [23].

We show for the first time in sheep, epigenetic modifications of the DLK1/MEG imprinted cluster in mature adult liver of females exposed to post-weaning undernutrition. Genetic ablation of DLK1 in the myogenic lineage impairs muscle development and DLK1 knock-out increases adiposity [34], [35]. In addition, an endocrine role of hepatic DLK1 has been suggested since specific manipulation of hepatic DLK1 expression can influence adipose tissue mass [50]. Although hepatic DLK1 expression was too low to measure in this study, it may be possible that the reduction in hepatic genes regulated by the DLK1/MEG3 imprinted cluster expression in females undernourished post-weaning is associated with reduced muscle mass and altered adipocyte development. Persistence of these effects in females may also negatively impact on metabolic homeostasis in the longer term.

These effects of post-weaning undernutrition on glucose metabolism were similar in females with or without prior exposure to early gestation undernutrition. However, in mature adult females exposed to post-weaning undernutrition without gestational undernutrition (CU group), adipose tissue and/or liver triglyceride handling was more sensitive to insulin, as given by a greater fall in TAG output during the GTT. Given the lack of an effect of post-weaning undernutrition on insulin-induced inhibition of NEFA output from adipose tissue, this effect may be largely in the liver. The reduction in adipose tissue lipid content and trend for enhanced adipose GR mRNA levels in females suggest that the relative balance between stored and circulating lipids in adipose tissue and liver could be influenced specifically by exposure only to post-weaning undernutrition. The potential mechanisms involved in this effect remain to be elucidated. Taken together, our findings suggest that changes in both glucose and lipid metabolism may be employed in CU females to enhance body mass once nutrition has been restored to normal. Even in the absence of any current effects on body weight or fatness [22], the dysregulation of lipid handling we have observed following undernutrition in the post-weaning period may increase risk of obesity or dyslipidaemia in later life [24].

In contrast to the effects of post-weaning undernutrition, undernutrition in early gestation in mature adult females, as well as in males, was associated with reduced insulin sensitivity in adipose tissue, as revealed by the insulin-induced inhibition of lipolysis (NEFA response during GTT), suggesting that this period may also influence lipid metabolism and fat accumulation and/or dyslipidaemia in the longer term. With this strategy in place, females exposed to post-weaning as well as early gestation undernutrition (UU group) may not require further alterations in adipose tissue and/or liver insulin sensitivity, as seen in the CU group, for body weight recovery.

In male sheep, we proposed that early gestation rather than post-weaning undernutrition would have a greater impact on mechanisms regulating adiposity. Males may have a heightened sensitivity to nutrient restriction in early gestation, when their growth rate is faster than females [51], potentially causing them to make adaptations to enhance their ability to optimise growth and body composition in later nutritionally adequate times. We demonstrated previously that males exposed to early gestation undernutrition have a relatively faster growth rate in the first 12 postnatal weeks without any change in birth weight [21]. In the current study we found that insulin sensitivity in adipose tissue, as revealed by the insulin-induced inhibition of lipolysis (NEFA response during GTT), was reduced in mature adult males undernourished in early gestation alongside increases in LPL and GR mRNA levels. Although these molecular effects were small and may not be reflected in subsequent protein expression, they were also associated with faster early postnatal growth. In this cohort, we did not observe long-term effects of early nutrition on fat depth [22], although this measurement was limited to one subcutaneous region, nor was there an effect on actual lipid content in adipose tissue at 2.5 years. However, lipid content may be maintained by a balance between the actions of glucocorticoids and LPL. Changes in glucocorticoid sensitivity and the ability to hydrolyse and transport triglycerides in early gestation-undernourished males could lead to changes in fat distribution or fat deposition in other organs such as the liver, effects that may be exacerbated by a subsequent abundant diet or with ageing. Indeed, the effect of early gestation undernutrition on adipose insulin sensitivity in males only became apparent at 2.5 years of age. The possibility of an altered plasma lipid profile in these animals, alongside our previous observations that exposure to early gestation undernutrition influences cardiac morphology and coronary function in adult male sheep [21], may contribute to a risk of cardiovascular disease. For female sheep exposed to early gestation undernutrition, the insulin-induced fall in NEFA during GTT was similar to that in males however there were no changes in LPL or GR mRNA levels in adipose tissue. While this may suggest these females do not have the same risk of dyslipidaemia, the implication of these differences requires further investigation. Our molecular studies of adipose tissue utilised the perirenal depot; given the differences in metabolic profiles between fat depots [52], the possibility that early gestation or post-weaning undernutrition influenced adipose tissue metabolism and/or deposition elsewhere in the body cannot be excluded from the current study.

In the current study, as well as examining the effects of both pre- and postnatal undernutrition on adult liver, we examined fetal liver in a separate cohort of sheep exposed to maternal undernutrition at 40% of control levels (compared to a 50% reduction in the study of adult offspring). Both left and right liver lobes were examined, except for the epigenetic analyses. There is unequal provision of umbilical venous blood between the liver lobes in fetal life, with predominantly more going to the left lobe, such that each lobe may be differentially sensitive to changes in maternal nutrient provision. Nutrient restriction in both early and late gestation reduced key gluconeogenic enzymes in late gestation liver and there was differential expression between the fetal liver lobes. These effects did not persist into adult life and indeed, as with the lobar differences in gene expression patterns, appear even to switch direction over the life course. We therefore show that the developing liver makes adaptations to reductions in maternal nutritional status and there is differential expression between the lobes. However, although these hepatic effects imply potential changes in gluconeogenic capacity, as well as growth potential [53], in response to maternal undernutrition, they may not translate to significant functional significance given the absence of changes in basal glucose in either exposed fetuses or adults [22], [40]. However, we must be cautious making direct comparisons between the fetal and adult cohorts reported here given the difference in the level of maternal nutritional challenge imposed. In addition, primiparous, singleton-bearing ewes were used in the fetal study, whereas older ewes carrying both singletons and twins were used in the adult study. Given findings that birth order poses a non-modifiable risk for obesity [54], the relative maternal constraint experienced by first born offspring may have imposed an additional environmental challenge to the fetuses exposed to maternal undernutrition.

The role of the liver as a sensor of maternal nutrient status is suggested in studies linking fetal liver blood flow with subsequent offspring fat mass [55], [56]. Experimental manipulation of fetal liver blood perfusion and reduction in maternal diet in sheep have both been shown in sheep to influence hepatic IGF expression [57], [58], with potential long term effects on offspring growth and metabolism. In addition, reduced DNA methylation at the IGF2/H19 imprinted locus has been reported in humans following the Dutch Famine [59] and in fetal and postnatal sheep in response to altered maternal diets [46], [60]. Unlike findings in lamb adrenal tissue [46], no effect of early gestation undernutrition was observed at the IGF2/H19 locus in adult sheep liver. However, methylation of IGF2R in liver of early gestation undernourished males was increased, which could increase IGF2R expression. Since this receptor acts to limit circulating IGF2 availability [61], this increase may influence growth potential or muscle growth. Although we were not able to demonstrate an effect of early gestation undernutrition on hepatic IGF2R gene expression, possibly because the affected CpG dinucleotides were not contiguous, others have also shown sex-specific effects on IGF2R expression and muscle area in mature cattle exposed to a maternal low protein diet in the first trimester [62]. Whether methylation patterns of these growth factors during early life were involved in control of lean *vs.* fat mass and growth patterns prior to tissue collection at 2.5 years remains unknown.

The current study did not identify any mechanisms that would enable male sheep exposed to post-weaning undernutrition to catch up once nutrition was restored to normal. The absence of any effects of undernutrition in this period on glucose tolerance and peripheral insulin sensitivity suggests that these males must employ other mechanisms to regain body weight following the nutritional challenge. All sheep were allowed access to the same food rations after the nutritional challenge but feed intake of individual animals was not determined when group-housed. It remains possible that the regulation of appetite was influenced by the post-weaning nutrient challenge [63].

Although we [22] and others [16] have shown no influence of the early gestation nutritional environment or being a twin on whole body glucose tolerance, we observed here some differences between singleton and twin offspring e.g. glucose transporter 4 mRNA levels in muscle, that were increased following early gestation undernutrition but only in twins. Twins face an additional nutritional constraint during gestation, with lower fetal glucose levels in late gestation and reduced birth weight [64] suggesting that the double insult of being a twin and facing gestational undernutrition may be required to induce such an effect [65]. Enhanced glucose uptake in muscle of these animals may contribute to the increased insulin sensitivity observed in twins in this cohort [22]. For singleton offspring, the strongest influence on glucose transporter 4 expression was nutrient restriction in the post-weaning period.

In conclusion, undernutrition during development induces changes in mechanisms of lipid and glucose metabolism which differ between tissues and sexes contingent on the period of nutritional restriction. Such changes may increase later life obesity and dyslipidaemia risk.

## Supporting Information

Figure S1 Flow diagram showing the total number of ewes and their offspring used in the study of adult sheep exposed to early gestation and/or post-weaning undernutrition.
(DOCX)

Table S1 Target gene primer and probe cDNA sequences for real-time PCR, methylation-sensitive PCR and pyrosequencing.
(DOCX)
