# Supplementary material for: Differential Pathways to Adult Metabolic Dysfunction following Poor Nutrition at Two Critical Developmental Periods in Sheep
Source: PLoS One. 2014 Mar 6;9(3):e90994. doi: 10.1371/journal.pone.0090994 (PMC3946277; doi:10.1371/journal.pone.0090994)
Supplement: Figure S1 — Flow diagram showing the total number of ewes and their offspring used in the study of adult sheep exposed to early gestation and/or post-weaning undernutrition. (DOCX) [file pone.0090994.s001.docx]

## Offspring allocated to post-weaning dietary groups (C or U)

## Ewes allocated to maternal dietary groups (C or U)

Welsh Mountain ewes (*n* = 59)

Restricted (U) nutrition in early gestation (*n* = 30)

● Ewes carrying singleton fetuses (*n* = 15)

● Ewes carrying twin fetuses (*n* = 15)

Control (C) nutrition in early gestation (*n* = 29)

● Ewes carrying singleton fetuses (*n* = 12)

● Ewes carrying twin fetuses (*n* = 17)

UU (*n* = 13)

● Males (*n* = 6)

● Females (*n* = 7)

UC (*n* = 12)

● Males (*n* = 4)

● Females (*n* = 8)

UU (*n* = 9)

● Males (*n* = 5)

● Females (*n* = 4)

UC (*n* = 6)

● Males (*n* = 4)

● Females (*n* = 2)

CC (*n* = 15)

● Males (*n* = 8)

● Females (*n* = 7)

CU (*n* = 10)

● Males (*n* = 5)

● Females (*n* = 5)

CU (*n* = 5)

● Males (*n* = 3)

● Females (*n* = 2)

CC (*n* = 7)

● Males (*n* = 5)

● Females (*n* = 2)

Twin offspring (*n* = 30)

● Twins studied as adults (*n* = 25)

● Twins not studied (*n* = 5)

Singleton offspring (*n* = 15)

Twin offspring (*n* = 34)

● Twins studied as adults (*n* = 25)

● Twins not studied (*n* = 9)

Singleton offspring (*n* = 12)
