# Supplementary material for: Differential Pathways to Adult Metabolic Dysfunction following Poor Nutrition at Two Critical Developmental Periods in Sheep
Source: PLoS One. 2014 Mar 6;9(3):e90994. doi: 10.1371/journal.pone.0090994 (PMC3946277; doi:10.1371/journal.pone.0090994)
Supplement: Table S1 — Target gene primer and probe cDNA sequences for real-time PCR, methylation-sensitive PCR and pyrosequencing. (DOCX) [file pone.0090994.s002.docx]

**Table S1 Target gene primer and probe cDNA sequences for real-time PCR, methylation-sensitive PCR and pyrosequencing**

| **Gene** | **Primers/Probe** | **Sequence for real-time PCR** |
| --- | --- | --- |
|  |  |  |
| IR | Forward primer | ACCGCCAAGGGCAAGAC |
|  | Reverse primer | AGCACCGCTCCACAAACTG |
|  | Probe | AACTGCCCTGCCACTGTCATCAACG |
| PEPCK | Forward primer | GATTGGCATCGAGCTGACAGA |
|  | Reverse primer | CGCCCATCCTCGTCATG |
|  | Probe | TCGCCCTACGTGGTGACCAGCA |
| G6Pase | Forward primer | TGGAGTCTTTTCAGGCATTGC |
|  | Reverse primer | CTTGAGACTGGCATTGTAGATGCT |
|  | Probe | TTGCTGAGACTTTCCGCCACATCCA |
| GLUT4 | Forward primer | CCGTGGCAGGACATTTGAC |
|  | Reverse primer | TTCCTGCTCCAGAAGAGAAGGT |
|  | Probe | ATCTCAGCCGTCTTCCGCCGG |
| LPL | Forward primer | ACCAGACTCCAACGTCATCGT |
|  | Reverse primer | GCTTGGTGTACCCTGCAGACA |
|  | Probe | TCACGGGCCCAGCAGCATTATCC |
| PPARγ | Forward primer | CAGCTCCGTGGACCTTTCTATG |
|  | Reverse primer | CGGTGGTGAAGGGCTTGAT |
|  | Probe | TGGATGACCACTCCCATGCTTTTGA |
| GR (liver) | Forward primer | ACTGCCCCAAGTGAAAACAGA |
|  | Reverse primer | GCCCAGTTTCTCCTGCTTAATTAC |
|  | Probe | AGAAGATTTTATCGAACTCTGCACCCCTGG |
| GR (adipose) | Forward primer | ACTGCCCCAAGTGAAAACAGA |
|  | Reverse primer | ATGAACAGAAATGGCAGACATT |
| IGF2R | Forward primer | GAGACACTTGTCCTCCGGAAAC |
|  | Reverse primer | CTCCTCGTAGCTCTTCCCATTG |
| IGF2 [[46](#_ENREF_46)] | Forward primer | GCTTCTTGCCTTCTTGGCCTT |
|  | Reverse primer | TCGGTTTATGCGGCTGGAT |
|  |  |  |
| **Gene** | **Primers** | **Sequence for methylation-sensitive PCR** |
|  |  |  |
| GR (liver) | Forward primer | CCACCTTTCCCCGAGTCACA |
|  | Forward control | CATGTGGGATCTGGTTTCTTGA |
|  | Reverse primer | CAATGCGTTGCTCACCATTT |
|  | Reverse control | CACCTGAGTACTCCTTCCTAAAAAAATT |
|  |  |  |
| **Gene** | **Primers/Probe** | **Sequence for pyrosequencing** |
|  |  |  |
| IGF2R | Forward primer | TGGAGTGTTTATAGAAAGAGGAGTAG |
|  | Reverse primer | CCTTCTCAACACCTTCACTCA |
|  | Probe | GGGTTTTTTTTTTGGTGAA |
| H19 | Forward primer | GGTGTTGGGGATGATAGG |
|  | Reverse primer | AAACTCCCACATTTAACCATAATC |
|  | Probe | GGGTGTGGAGATAGA |
| MEG3 | Forward primer | GTGGTGGGTTTATAAATAGTTTAGT |
|  | Reverse primer | ATCAAATTAAAAAAAACTCCCATACTCTT |
|  | Probe | ATAGTTTTATAGTTATTTATAGGAT |
|  |  |  |
